# Urban–Rural Disparities in Access to Low-Dose Computed Tomography Lung Cancer Screening in Missouri and Illinois

**DOI:** 10.5888/pcd17.200202

**Published:** 2020-11-05

**Authors:** Karthik W. Rohatgi, Christine M. Marx, Marquita W. Lewis-Thames, Jingxia Liu, Graham A. Colditz, Aimee S. James

**Affiliations:** 1 Division of Public Health Sciences, Department of Surgery, Washington University School of Medicine, St. Louis, Missouri; 2 Department of Medical Social Sciences, Center for Community Health, Northwestern University Feinberg School of Medicine, Chicago, Illinois

## Abstract

**Introduction:**

Low-dose computed tomography (LDCT) lung cancer screening is recommended for current and former smokers who meet eligibility criteria. Few studies have quantitatively examined disparities in access to LDCT screening. The objective of this study was to examine relationships between 1) rurality, sociodemographic characteristics, and access to LDCT lung cancer screening and 2) screening access and lung cancer mortality.

**Methods:**

We used census block group and county-level data from Missouri and Illinois. We defined access to screening as presence of an accredited screening center within 30 miles of residence as of May 2019. We used mixed-effects logistic models for screening access and county-level multiple linear regression models for lung cancer mortality.

**Results:**

Approximately 97.6% of metropolitan residents had access to screening, compared with 41.0% of nonmetropolitan residents. After controlling for sociodemographic characteristics, the odds of having access to screening in rural areas were 17% of the odds in metropolitan areas (95% CI, 12%–26%). We observed no association between screening access and lung cancer mortality. Southeastern Missouri, a rural and impoverished area, had low levels of screening access, high smoking prevalence, and high lung cancer mortality.

**Conclusion:**

Although access to LDCT is lower in rural areas than in urban areas, lung cancer mortality in rural residents is multifactorial and cannot be explained by access alone. Targeted efforts to implement rural LDCT screening could reduce geographic disparities in access, although further research is needed to understand how increased access to screening could affect uptake and rural disparities in lung cancer mortality.

**Summary**

**What is already known on this topic?**

Low-dose computed tomography (LDCT) screening for lung cancer is recommended for current and former smokers meeting eligibility criteria. As of 2017, rural areas generally had less geographic access to LDCT screening than urban areas.

**What is added by this report?**

Despite the recent proliferation of LDCT screening, rural areas in Missouri and Illinois have low levels of access to screening. We observed no association between geographic access to screening and lung cancer mortality.

**What are the implications for public health practice?**

As LDCT screening becomes more widespread, future studies need to evaluate its effects on population-level lung cancer mortality rates in urban and rural areas.

## Introduction

Low-dose computed tomography (LDCT) screening has increased the ability to detect early-stage lung cancer in recent years (1). The National Lung Screening Trial showed that LDCT screening reduces risk of lung cancer death by up to 20%, compared with chest x-ray (1). In light of this evidence, the US Preventive Services Task Force (USPSTF) issued a recommendation to provide annual LDCT screening to adults aged 55 to 80 who have at least a 30 pack-year smoking history, currently smoke or quit in the past 15 years, and have no lung cancer symptoms (2). Medicare subsequently began reimbursing screening of adults aged 55 to 77 (2). Unique among cancer screenings, LDCT reimbursement is contingent on provision of smoking cessation counseling and shared decision making, both of which are also billable services (2).

The burden of these requirements on physician practices, along with the high rate (>95%) of false-positive test results (1), may explain why screening rates are low. Although the number of accredited LDCT centers nationwide increased from an estimated 203 in 2014 to 1,748 in early 2017 (3), a study of 10 geographically diverse US states found that 12.7% of adults aged 55 to 80 met USPSTF criteria for LDCT screening in 2017, but of these adults, only 12.5% reported receiving screening in the previous year (4).

Barriers to LDCT screening persist — rural residents nationwide have less access, defined as distance and driving time, to LDCT screening than their urban counterparts (3,5). Although more than 95% of adults aged 55 to 79 in 8 northeastern states (Connecticut, Delaware, Maryland, Massachusetts, New Jersey, New York, Pennsylvania, Rhode Island) have access to a screening center within 30 miles (Euclidean distance), the proportion in the Midwest (Illinois, Indiana, Iowa, Kansas, Michigan, Minnesota, Missouri, Nebraska, North Dakota, Ohio, South Dakota, Wisconsin) is lower and highly variable (22%–93%) (3).

Our investigation focused on Missouri and Illinois, both Midwestern states in the upper Mississippi Delta, a region marked by high cancer mortality (6). Missouri and Illinois are home to 6.1% of the US population and contain a heterogeneous mix of geographies, from densely populated cities to rural farmland. Both states reflect the nationwide pattern of higher smoking prevalence in rural areas than in urban areas (7).

The 2 states have significantly different policies on health care and tobacco. Illinois was an early expander of Medicaid under the Affordable Care Act, whereas Missouri was not. The state cigarette tax is more than 15 times higher in Illinois ($2.98/pack) than in Missouri ($0.17/pack) (8). Demographically, Missouri has a higher proportion of rural residents than the United States as a whole (29.6% for Missouri vs 19.3% nationwide), whereas Illinois, at 11.5%, has a lower proportion (9). A study published in 2018 identified Missouri as a state with moderate access to LDCT screening and high lung cancer mortality and Illinois as a state with high access to screening and moderate mortality (3).

Given rural–urban differences and the importance of using precise and localized estimates to drive public health priorities (10), we performed a detailed analysis of screening access in Missouri and Illinois. Efforts to reduce rural–urban disparities in LDCT screening and lung cancer mortality require county-specific information on screening “deserts” and mortality hotspots (6). As such, the primary objective of this study was to identify locations in Missouri and Illinois that have high lung cancer mortality and/or cigarette smoking rates but low levels of access to LDCT screening; these locations are priority areas for intervention. We built on previous work (5) by using multilevel, mixed-effects modeling to quantify the association between rurality, sociodemographic characteristics, and access to screening at the census block group level. Additionally, a secondary objective was to conduct an exploratory analysis of the relationship between access to screening and lung cancer mortality.

## Methods

### Data management

We collected and organized data by using methods similar to those of Eberth et al (3). In May 2019, we obtained addresses of screening centers accredited by the American College of Radiology (11) and Lung Cancer Alliance (now GO_2_ Foundation for Lung Cancer) Screening Centers of Excellence (12). We compiled addresses for 356 centers in Missouri, Illinois, and all neighboring states (Arkansas, Indiana, Iowa, Kansas, Kentucky, Michigan, Nebraska, Oklahoma, Tennessee, Wisconsin). We collected addresses from neighboring states because patients may cross state lines to reach the nearest center. When multiple screening centers were located on a single hospital campus, we randomly chose 1 center. Additionally, we removed from analysis 1 center in Indiana that was closed. We performed automatic geocoding in ArcGIS Desktop version 10.6 using the USA Geocoding Service (Esri). We used interactive rematch for screening centers that matched equally well to multiple street addresses.

We manually rematched all unmatched centers and centers matched to a zip code rather than a street address (n = 56 centers) by using a Google Maps API (application programming interface; https://developers.google.com/maps/documentation/geocoding/intro). Consistent with the methods of Eberth et al, we constructed a 30-mile planar buffer around each screening center to represent the area in which that center was deemed accessible (3). A nationwide study comparing driving distance and straight-line distance from all census tracts to the closest hospital found that the 2 measures are highly correlated in the absence of shorelines, mountains, or other physical barriers (13). Missouri and Illinois contain few such barriers; thus, we felt justified in using a 30-mile straight-line buffer. Hospital “deserts” are defined by the lack of a hospital within a 30-mile radius (14). Consistent with Eberth et al, we considered a center accessible to residents of census block groups whose centroids lay inside the buffer (3).

We used these data to calculate the county-wide percentage of residents aged 55 to 79 who have access to LDCT screening within 30 miles. We obtained census block group–level data on age from American Community Survey (ACS) 2013–2017 five-year estimates (15). Of the available categories, the age group 55 to 79 was the closest option to the recommended screening age range of 55 to 80 (15).

### Measures

**Screening access measure.** We dichotomized access to LDCT screening at the census block group–level as presence or absence of at least 1 center within 30 miles of the centroid. At the county level, we quantified access by the proportion of adults aged 55 to 79 who lived in a census block group and met this criterion. Because appropriate data on smoking status were unavailable, we assumed that the ratio of adults aged 55 to 79 to LDCT-eligible adults was roughly constant across all census block groups in a county.

**Rurality measures.** We used census tract–level rural–urban commuting area (RUCA) codes to measure rurality (16). For modeling purposes, we grouped codes 1 to 3 as metropolitan, codes 4 to 6 as micropolitan, and codes 7 to 10 as small town/rural areas. However, because lung cancer mortality data were available only at the county level, we used the National Center for Health Statistics (NCHS) county-level classification (17) for our exploratory mortality model. NCHS codes range from 1 (large central metro) to 6 (noncore). We used RUCA codes for our main access model because they provide more fine-grained information than NCHS codes on rurality in a census tract and its census block groups.

**Sociodemographic characteristics.** We obtained demographic census block group–level data from ACS 2013–2017 five-year estimates (15). We defined income as median annual household income (in thousands of dollars), education as percentage of residents aged 25 or older with at least a college degree, and race as the percentage of White residents and the percentage of African American residents.

**Lung cancer and smoking measures.** We obtained county-level, age-adjusted lung and bronchus cancer mortality rates during 2013–2017 from the National Cancer Institute’s Surveillance, Epidemiology, and End Results (SEER) program via SEER*Stat software version 8.3.6 (18). We used mortality rates (per 100,000) for people aged 60 or older. Given the lead-time bias and additional survival time after lung cancer diagnosis, we believed mortality in this age range was most likely to be affected by a screening program for people aged 55 to 80. We suppressed data from 1 county in Missouri because of a small number (<10) of deaths. We obtained data on 2019 adult smoking prevalence from County Health Rankings (19). We classified adults as smokers if they reported currently smoking every day or most days and having smoked at least 100 cigarettes in their lifetime.

**Map development. **We obtained census block group shapefiles from the Census Bureau (15) and state-level and county-level shapefiles from Esri (20). We created categories by rounding quintiles to the nearest 10% for access to screening, nearest 10 per 100,000 for lung cancer mortality, and nearest 0.5% for smoking prevalence. Mortality and smoking quintiles were based on national (rather than bi-state) data, to emphasize how Illinois and Missouri compare with other states. We created maps in ArcGIS Desktop version 10.6 (Esri).

### Statistical analysis

For the first analysis, our outcome of interest was access to screening within 30 miles of the census block group centroid (binary). Predictor variables were rurality as quantified by RUCA codes (main predictor; categorical), income (continuous), education (continuous), and race (continuous). We used multilevel, mixed-effects logistic regression modeling to determine the association between outcome and predictor variables. In this model, the census block group was the unit of analysis. We defined RUCA codes at the census tract level; all other variables were defined at the census block group level.

Our modeling procedure was as follows: first, we considered bivariate logistic models to examine crude associations between screening access and each predictor. We then used the full additive model with all predictor variables (fixed effects) and random intercepts for each state and county. Counties were nested within states. Census tract was not considered a random effect because of the small number of census block groups in some tracts. We then tested models involving interaction terms and random slopes for various predictors. These terms were all nonsignificant and thus not included in the final model. We calculated the odds ratio (OR), 95% CI, and *P* value associated with each fixed-effect parameter.

Our second, exploratory model used the county as the unit of analysis. We sought to determine the association between access to LDCT screening, defined as the proportion of residents aged 55 to 79 whose census block group of residence is located within 30 miles of a screening center (main predictor), and lung cancer mortality rate in adults aged 60 or older (outcome). Other covariates included adult smoking prevalence, rurality (NCHS code), income, education, race, and state in which the county is located. We used multiple linear regression modeling for this county-level analysis. We defined all variables at the county level, and all variables except NCHS code were continuous. Because only 3 counties in the study area were designated as large central metro (level 1), we performed a sensitivity analysis using a dichotomized rurality variable (levels 1–4 [all metro area counties] vs levels 5–6 [micropolitan and noncore]).

For both analyses, all tests were 2-sided and *P* < .05 was considered significant. We calculated variance inflation factors to assess evidence of multicollinearity. For the main mixed-effects model, we assessed county-level random intercepts for normality. For the multiple regression model, we checked residual plots for normality and constant variance. We performed statistical analyses in R version 3.6.1 (The R Project for Statistical Computing).

## Results

**Figure 1:**
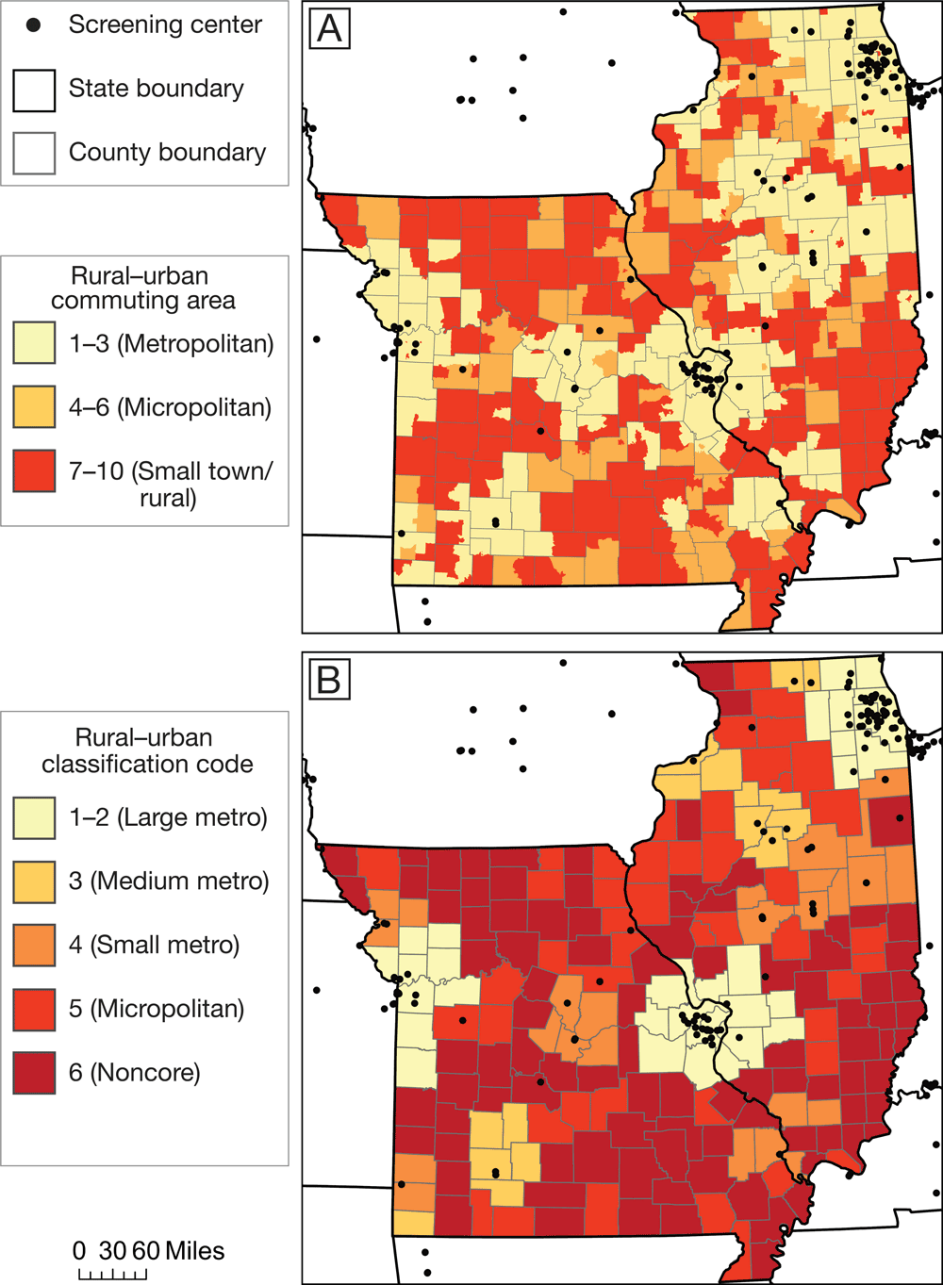
Measures of rurality in Missouri and Illinois and location of low-dose computed tomography screening centers. A, Rural–urban commuting area (RUCA) categories at the census tract level, determined by US Department of Agriculture Economic Research Service (16). B, National Center for Health Statistics (NCHS) rural–urban classification codes at the county level (17). Data on screening centers obtained from American College of Radiology (11) and GO_2_ Foundation for Lung Cancer (12). Shapefiles obtained from ESRI (20).

**Figure 2:**
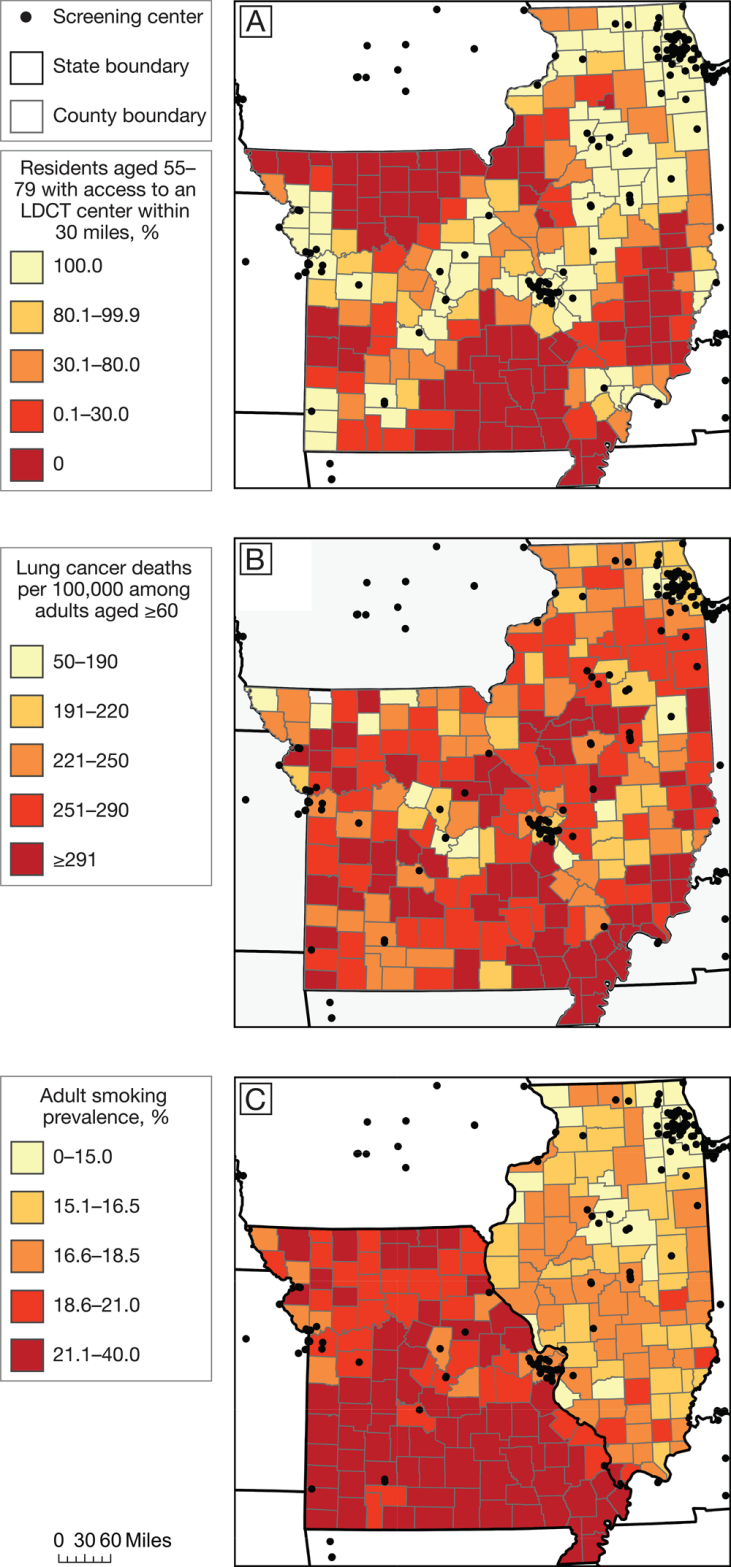
Access to LDCT lung cancer screening, lung cancer mortality, and smoking prevalence in Missouri and Illinois. A, Percentage of residents aged 55–79 with access to an LDCT lung cancer screening center within 30 miles. B, Lung cancer mortality (deaths per 100,000) among adults aged ≥60. C, Adult smoking prevalence. All maps are at the county level, and categories are based on rounded quintiles. Data obtained from American College of Radiology (11), GO_2_ Foundation for Lung Cancer (12), Surveillance, Epidemiology, and End Results program (18), and County Health Rankings (19). Shapefiles from ESRI (20). Abbreviation: LDCT, low-dose computed tomography.

Overall, 91.2% of Illinois residents aged 55 to 79 and 78.3% of their Missouri counterparts were within 30 miles of an LDCT screening center. Areas with low access to screening corresponded roughly to the states’ most rural regions (Figure 1). These areas of low access included central northern Missouri, the Bootheel region in southeastern Missouri, and southern Illinois (Figure 2A). LDCT screening centers in Illinois and Missouri were located in census block groups whose residents were more likely than residents in the 2-state region as a whole to identify as White (76.6% vs 67.6%) and have at least a college degree (45.1% vs 31.8%). Similarly, weighted median income in census block groups containing screening centers was $72,222, compared with $57,750 across all census block groups.

Both states had pockets of high lung cancer mortality, although smoking rates were consistently higher in Missouri than in Illinois (Figure 2B and 2C). Southeastern Missouri had the highest concentration of both lung cancer mortality and adult smokers.

In metropolitan area cores or nearby commuting areas (RUCA codes 1–3), 97.6% of residents had access to LDCT screening, compared with 41.0% of residents in micropolitan or small town/rural areas (codes 4–10). This difference in access was similar across NCHS county-level codes (Table 1). Furthermore, as rurality increased, we observed higher rates of adult smoking and lung cancer mortality among adults aged 60 or older.

**Table 1:** Lung Cancer Screening Access Within 30 Miles, Adult Smoking Prevalence, and Age-Adjusted Lung Cancer Mortality, by Urban–Rural Designations, Missouri and Illinois, 2013–2019

Urban–Rural Designation^a^	No. of Counties	Population Aged 55–79, N (%)^b^	Population With Screening Access, %^c^	Adult Smoking Prevalence, %^d^	Age-Adjusted Lung Cancer Mortality Among Residents Aged ≥60^e^
Large central metro	3	1,378,581 (30.6)	100.0	15.2	214
Large fringe metro	30	1,524,652 (33.8)	98.6	16.0	226
Medium metro	16	351,843 (7.8)	96.4	18.4	244
Small metro	25	416,522 (9.2)	89.3	18.2	250
Micropolitan	46	418,276 (9.3)	42.8	19.2	269
Noncore	97	421,917 (9.4)	34.9	20.0	277

a Determined by National Center for Health Statistics (17).

b Based on 2013–2017 data (15).

c Based on 2019 data on screening center location (11,12). Proportion of population whose census block group of residence is within 30 miles of a screening center; computed as averages of county-level data weighted by number of residents aged 55–79 (as of 2013–2017).

d Based on 2019 data (19). Proportion of adults who currently smoke and have smoked ≥100 cigarettes in their lifetime; computed as averages of county-level data weighted by number of adult residents (as of 2013–2017).

e Based on 2013–2017 data (18). Rate per 100,000 population; computed as averages of county-level data weighted by number of residents aged ≥60 (as of 2013–2017).

The mixed-effects logistic regression model of access to LDCT screening within a 30-mile radius achieved convergence, and a likelihood ratio test showed that inclusion of random effects significantly improved fit (χ^2^ = 3417.6; *df* = 2; *P* < .001). Small town and rural census block groups had significantly lower adjusted odds than metropolitan census block groups of access to screening within a 30-mile radius (OR = 0.17; 95% CI, 0.12–0.26) (Table 2). Screening access in micropolitan areas was similarly lower than in metropolitan areas (OR = 0.17; 95% CI, 0.10–0.27).

**Table 2:** Census Block Group–Level (N = 13,834 Census Block Groups) Association Between Degree of Rurality (in 2019) and Access to Lung Cancer Screening Within 30 Miles (in 2019) Adjusted for Demographic Characteristics, Missouri And Illinois, 2013–2017

Model Parameter	Unadjusted Model		Adjusted Model	
	OR (95% CI)	*P* Value	OR (95% CI)	*P* Value
**Degree of rurality^a^**				
Metropolitan (RUCA codes 1–3)	1.00 [Reference]		1.00 [Reference]	
Micropolitan (RUCA codes 4–6)	0.019 (0.016–0.022)	<.001	0.17 (0.10–0.27)	<.001
Small town or rural (RUCA codes 7–10)	0.017 (0.015–0.020)	<.001	0.17 (0.12–0.26)	<.001
**Demographic characteristics^b^**				
Median annual household income, in thousands, $^c^	1.03 (1.03–1.03)	<.001	1.01 (1.00–1.02)	.09
Percentage of population aged ≥25 with a college degree^d^	1.05 (1.05–1.06)	<.001	1.01 (1.00–1.03)	.08
Percentage of population that is White^d^	0.91 (0.91–0.92)	<.001	1.02 (1.00–1.03)	.05
Percentage of population that is African American^d^	0.95 (0.94–0.96)	<.001	1.01 (0.99–1.03)	.32

Abbreviations: OR, odds ratio; RUCA, rural–urban commuting area.

a Census tract–level RUCA codes used to measure rurality (16).

b Determined by American Community Survey 5-year estimates (15).

c Odds ratio represents $1,000 increase in median annual household income.

d Odds ratio represents 1 percentage-point increase in the corresponding variable.

In the county-level models, we found no significant relationship between access to LDCT screening and lung cancer mortality after adjusting for smoking prevalence, rurality, and demographic characteristics (*P* = .68) (Table 3). The variables most strongly associated with lung cancer mortality per 100,000 residents were smoking prevalence (β = 9.7; 95% CI, 4.6 to 14.9), percentage of population aged 25 or older with a college degree (β = −2.7; 95% CI, −1.5 to −3.9), and residence in Missouri (β = −41.2; 95% CI, −68.2 to −14.2). Thus, a 1 percentage-point increase in smoking prevalence was associated with a mortality increase of 9.7 per 100,000 residents, and a 1 percentage-point increase in the fraction of individuals aged 25 or older with a college degree was associated with a decrease of 2.7 per 100,000. Rurality and other variables showed no association, and use of a binary rurality variable (all metropolitan vs micropolitan/noncore) yielded nearly identical results.

**Table 3:** County-Level (N = 210 Counties) Association Between Proportion of Residents With Access to Screening Within 30 Miles (in 2019) and Age-Adjusted Lung Cancer Mortality Among Adults Aged ≥60 (in 2013–2017), Adjusted for Rurality (in 2019), and Demographic Characteristics (in 2013–2017), Missouri and Illinois

Model Parameter	Change in Mortality per 100,000 Population, β (95% CI) [*P* Value]
**Percentage of census block groups with access to lung cancer screening within 30 miles**	0.04 (−0.15 to 0.23) [.68]
**Degree of rurality^a^**	
Large central metro	1 [Reference]
Large fringe metro	8.9 (−54.8 to 72.6) [.78]
Medium metro	−8.7 (−74.5 to 57.0) [.79]
Small metro	3.4 (−58.3 to 65.2) [.91]
Micropolitan	2.7 (−60.9 to 66.3) [.93]
Noncore	−4.6 (−68.5 to 59.3) [.89]
**State**	
Illinois	1 [Reference]
Missouri	−41.2 (−68.2 to −14.2) [.003]
**Demographic characteristics**	
Percentage of population that reports smoking^b^	9.7 (4.6 to 14.9) [<.001]
Median annual household income, in thousands, $^c^	0.4 (−0.9 to 1.8) [.52]
Percentage of population aged ≥25 with a college degree^c^	−2.7 (−3.9 to −1.5) [<.001]
Percentage of population that is White^c^	0.2 (−1.1 to 1.6) [.76]
Percentage of population that is African American^c^	0.8 (−1.1 to 2.7) [.42]

a Determined by National Center for Health Statistics (17).

b Determined by 2019 County Health Rankings (19).

c Determined by American Community Survey 5-year estimates (15).

## Discussion

Our study examined access to LDCT screening across diverse urban and rural areas, and in communities of varying sociodemographics. The odds of urban populations having access to screening were more than 5 times greater than those of micropolitan or rural counterparts. After adjusting for smoking prevalence and demographic characteristics, we found no evidence that greater access to screening or greater urbanization is associated with lower county-level lung cancer mortality. However, counties with a larger proportion of college-educated residents or lower smoking prevalence tended to have lower lung cancer mortality.

Several studies reported that rural residents have lower access to LDCT screening (3,5,21), and our study confirms those findings. Our study also found that micropolitan areas have no better access than rural areas. Findings from our study reveal a negligible association between access to LDCT screening and lung cancer mortality rates.

Most likely, the observed lack of association between access to screening and mortality was due to the nascent state of LDCT screening and low uptake during the years of mortality data used in our study (2013–2017) (4). Screening can detect early-stage and slow-growing cancers that would not have otherwise been diagnosed for quite some time. Because lung cancer tends to be diagnosed at late stages with poor survival rates, several years of higher rates of screening may be needed before reduced mortality is seen. The overall delay from screening implementation to decrease in mortality roughly equals the sum of lead-time bias (approximately 1–3 years for LDCT) (22) and the traditional (without screening) survival time. Other variables may have affected our mortality analysis. In Illinois, a major coal-producing state, residential proximity to coal mines is associated with increased lung cancer incidence and mortality (23). Regardless, our analysis represents valuable baseline research and demonstrates the importance of attending to county-level disparities. An increase in LDCT screening uptake would likely reduce lung cancer mortality at the population level. On the basis of colorectal cancer screening research, we believe that greater geographic access to LDCT screening could effectively increase uptake (24). Improving geographic access to a service with low uptake is still worthwhile, because poor access may be contributing to low uptake.

Although rural areas are associated with poorer health outcomes than urban areas (25), we must also consider the urban–rural paradox, which suggests that among urban residents, greater distance to health care facilities is inversely associated with receiving care, but among rural residents, greater distance is positively associated with receiving care (26). Using 2015 data, Odahowski et al found that LDCT screening uptake was similar across metropolitan and nonmetropolitan counties, although low rates in both areas (<4%) make it difficult to understand why uptake is similar and whether the similarity will be maintained over time (27). The similarity in screening uptake rates may result from selection bias: the few people who completed screening may be the most enthusiastic and well-resourced patients in both urban and rural areas. Increased geographic access to LDCT screening may be needed to further increase uptake in rural areas. Further studies using discriminate, comprehensive measures of access and uptake are needed to explore whether geographic availability of screening has a different effect on mortality in urban and rural areas.

Previous research on geographic access to LDCT screening is minimal. To our knowledge, ours is the first study to assess access to LDCT screening, associated demographic determinants, and implications for mortality at a local population level. However, our study has several limitations. First, limited availability of public data necessitated the use of variables from 2 different periods. Demographic and lung cancer mortality data were from 2013–2017, whereas data on smoking prevalence and access to screening were from 2019. Second, we used data from multiple sources, including telephone surveys, online surveys, and government registries. Each source has its own limitations and can contribute to biased model estimates. Third, the ecological study design based on census block group–level and county-level data precludes extensive application of our conclusions about the relationships between rurality, access, and mortality to any 1 person. Fourth, in our exploratory analysis, county-level rates of access to LDCT screening were based on all residents aged 55 to 79, regardless of smoking status or other screening eligibility criteria. By taking this approach, we assumed that the percentage of residents aged 55 to 79 who meet eligibility criteria was roughly constant within a county; we made no assumptions about differences between counties. Finally, we included in our analyses only GO_2_ Foundation Screening Centers of Excellence and American College of Radiology accredited centers. Thus, our analyses may have underestimated the proportion of residents, especially in rural areas, who had access to some form of screening. However, accredited LDCT programs may deliver a better level of care than nonaccredited programs (28).

This study underscores the need for further research and creative solutions for increasing LDCT screening in rural areas, especially in the Mississippi Delta region, where significant cancer disparities exist. Not doing so may propagate the urban–rural disparities that exist in other cancer screening programs, such as mammography (25). Further research may be especially important in areas with high rates of smoking and lung cancer mortality, such as southeastern Missouri. In the past few years, mobile LDCT screening has been introduced in dozens of rural communities in Georgia and Tennessee (29). Incorporation of telemedicine could also circumvent the difficulty of finding qualified on-site specialists to interpret LDCT scans and recommend treatment in rural areas. Teleradiology is now a ubiquitous practice, allowing radiologists to bill for LDCT and other interpretations furnished off-site. Some teleoncology programs offer remote interpretation of biopsy specimens (30), which is occasionally required as a follow-up to LDCT screening. Additionally, screening must be coupled with effective smoking cessation interventions to maximize reductions in mortality.

Finally, our results emphasize the need for data-driven, locally targeted programs to increase screening and decrease mortality. In Missouri and Illinois, many areas with high rates of smoking and lung cancer mortality have low access to screening. However, some areas with high rates of smoking and lung cancer mortality, such as the rural counties north of Kansas City, have good access to screening. State or national one-size-fits-all programs to simply add more screening centers may not be helpful in these communities.

Our study adds to the growing body of evidence on urban–rural disparities in access to screening, while exploring the effects of access to LDCT screening on lung cancer mortality. County-specific approaches are needed to increase access to screening in rural areas with high mortality. At the same time, further implementation research is needed to understand how to effectively minimize individual and system-level barriers to rural screening.
