# Erratum, Vol. 17, November 5 Release

**DOI:** 10.5888/pcd19.200202e

**Published:** 2022-04-07

The maps in Figure 2A and 2B were incorrect in the article “Urban–Rural Disparities in Access to Low-Dose Computed Tomography Lung Cancer Screening in Missouri and Illinois.” The correct maps were inserted and posted on March 23, 2022: https://www.cdc.gov/pcd/issues/2020/20_0202.htm . We regret any confusion this error may have caused.

